# Introduction to the Development of Skin in Vertebrates

**DOI:** 10.3390/jdb11010007

**Published:** 2023-01-31

**Authors:** Lorenzo Alibardi

**Affiliations:** 1 Comparative Histolab Padova, 35100 Padova, Italy; lorenzo.alibardi@unibo.it; 2 Department of Biology, University of Bologna, 40126 Bologna, Italy

## 1. Background

The integument of vertebrates is a complex and large organ positioned at the interface with the aquatic or terrestrial environment, and is derived from the embryonic ectoderm (epidermis) and mesoderm (dermis and hypodermis). During piscine to land vertebrate evolution, the development of specific characteristics in both the epidermis and dermis allowed these vertebrates an almost perfect adaptation to their environment [1,2,3,4]. In fish and most amphibians (anamiotes), keratinization is the prevalent form of terminal keratinocytes differentiation, and the largest amount of proteins that are accumulated in these cells are intermediate filament keratins (IF-keratins or alpha-keratins). In amniotes (reptiles, birds, and mammals) epidermis, in addition to IF-keratins, a variable process of soft or hard cornification takes place, which differs in intensity in different integument appendages, such as scales, feathers, hairs, claws, horns, etc. [5,6,7]. Cornification derives from the addition of numerous corneous proteins to the initial meshwork of IF-keratins present in keratinocytes, and corneous proteins can even surpass the amount of IF-keratins in fully mature corneocytes. The keratin-associated corneous proteins are generated in different genome loci from those coding for IF-keratins, and the largest locus only present in amniotes is represented by the EDC (epidermal differentiation complex) [8]. The epidermis is also one of the main immune organs as it contains numerous immune cells that build the initial anti-microbial barrier, and it is particularly active in terrestrial vertebrates [9]. Additionally, specific receptors are differentiated in the skin of anamniotes and amniotes, and they allow sensing the external environmental conditions for an effective vertebrate response.

In the dermis and hypodermis of diverse vertebrates, a variable collection of fibroblasts, chromatophores, immune cells, lipoblasts, blood vessels, nerves, and other cell types are accumulated, allowing specific skin functions in the aquatic or land environment. In fish and sparse amphibians, reptiles, and mammals the dermis is also colonized by osteogenic cells capable of forming bony scales (fish) or osteoderms (tetrapods), structures that mechanically strengthen the skin [10,11].

The definitive integument derives from successive morphogentic steps that mainly occur during the last part of embryogenesis or even in post-embryonic stages in vertebrates after most of the internal organs have formed in the embryo. This allows the new individual to face the aquatic or land environment with an initial skin adaptation. The presentation of representative examples of skin development from the wide variety of vertebrate groups is the principal goal of this Special Issue.

## 2. Topics Presented in the *JDB* Special Issue

This Special Issue of the *Journal of Developmental Biology* on the “Development of the skin in vertebrates” aims to summarize some comparative morphological and cell biology information on skin formation, the covering organ that interfaces with the liquid, humid, and dry environment, in all vertebrate classes. It includes various contributions from researchers that actively study the skin development of fish and mammals in different countries, providing an updated comparative and synthetic view of the evolution of the skin and its appendages in vertebrates adapted to diverse ecological conditions. This Special Issue first provides a general introduction on the structure and development of the integument in vertebrates, centered on the developmental origin of the skin, which includes the specific adaptations that are initially developed in the skin of amniotes (fish and amphibians) and later in terrestrial vertebrates (amniotes). The following chapter deals with the developmental aspects of the skin in amphibians, the anamniotes with an aquatic adaptation during their initial phase of life before later transiting to the land. The next chapters deal with an analysis of the formation of the skin and its mechanical and sensorial appendages in fully terrestrial vertebrates, including the evolution and diversification of the corneous layer of the epidermis in reptiles, birds, and mammals, as well as the differentiation of their specific skin appendages, particularly scales, feathers, and hairs. The passage from an aquatic–semiaquatic (fish–amphibian) to terrestrial vertebrate (sauropsid and mammals) remains partly detectable during development in amniotes by the study of the stratum corneum’ evolution. This collection of papers should provide a broad and updated summary of the present knowledge on the formation of the vital integuments of vertebrates, which allow them to live in their specific habitats.
